# Design and Monitoring Application of an Adjustable Intelligent Bearing Based on Pressure Sensing

**DOI:** 10.3390/s24237820

**Published:** 2024-12-06

**Authors:** Shu Li, Zaiyu Zhang, Luyi Gan, Jiheng Yin, Ming Fu

**Affiliations:** 1 School of Safety Science and Engineering, Anhui University of Science and Technology, Huainan 232001, China; lishu@tsinghua-hf.edu.cn (S.L.); zhangyouduowen@163.com (Z.Z.); 2 Hefei Institute for Public Safety Research, Tsinghua University, Hefei 230601, China

**Keywords:** structural health monitoring, bridge overturning, intelligent sensors, bearing reaction force

## Abstract

Single-pier, dual-bearing bridges are susceptible to effects such as concrete creep, thermal expansion, and uneven foundation settlement. When combined with eccentric loading from heavy vehicles, these factors collectively can significantly increase the risk of bridge overturning. To address this risk, a comprehensive analysis of the bridge overturning mechanism was conducted. Considering the current limitations of health monitoring in bearing reaction force (BRF) measurement and risk mitigation, an adjustable intelligent bearing based on pressure sensing and self-locking principles was developed. Its mechanical performance was analyzed under the most unfavorable load conditions. To further validate the approach, a specific experimental bridge was used as a case study. The effectiveness of the force measurement and height adjustment functions was evaluated through moving load experiments. The results showed that the force measurement function accurately captured dynamic BRF changes within a precision range of ±0.1% FS and demonstrated high sensitivity to instantaneous impact effects. The height adjustment function achieved a reaction force change of up to 40 kN within the maximum adjustment range of 1.2 mm, significantly improving the load distribution of the bridge. These findings validated the reliability of the proposed intelligent bearing in real-time monitoring and proactive risk adjustment. This effectively overcomes the limitations of existing bearings, which only perform passive monitoring. Overall, it achieves the real-time monitoring of BRF and proactive control of bridge overturning risks.

## 1. Introduction

In recent years, with advancements in technology and infrastructure development, structural health monitoring (SHM) has gained widespread application [1]. By deploying sensors on the bridge, SHM can monitor the stress state, deflection, and environmental parameters in real time. This helps to identify potential structural issues promptly, enabling management to take effective preventive measures [2]. In this context, vibration-based real-time eigen perturbation techniques have been extensively studied. These methods utilize the variations in the dynamic characteristics of vibrating systems. They are employed for modal identification and damage diagnosis. Such techniques demonstrate unique advantages in evaluating the long-term performance of structures [3,4]. Furthermore, analyzing and assessing long-term monitoring data provides a scientific basis for daily maintenance and condition evaluation. SHM has become an indispensable component of modern bridge safety management [5].

Currently, SHM research primarily focuses on changes in beam structures. It typically evaluates the working condition and health status of beams by monitoring real-time variations in stress, strain, and deflection. For example, some common monitoring methods include installing resistive strain sensors and fiber Bragg grating strain sensors at key cross-sections of the main beam. These sensors capture the stress–strain response under different load conditions. This helps assess the ultimate strength of the bridge [6,7]. Additionally, sensors such as static leveling instruments, displacement monitors, and inclinometers can track spatial deformation and displacement changes of the main beam during operation. This helps reflect changes in the beam’s stiffness. It also assists in determining the presence of structural fatigue or local damage [8,9]. However, in actual bridge design, the main beam is assigned sufficient strength and redundancy. This reduces the risk of fracture. In contrast, the risks of overturning and beam dropping are often more critical. Currently, health monitoring systems still lack adequate monitoring sensors for these key boundary nodes. This makes it difficult to effectively prevent and control related risks [10].

On the other hand, the risks associated with bridge structures are often sudden and unpredictable. This is especially true in cases of extreme weather or unexpected traffic overload, where the speed of risk occurrence is very rapid [11]. Feng et al. [12] proposed a method to evaluate the rotational stiffness of bridge supports using axle acceleration. They introduced a transfer function model that is easy to calibrate. This method overcomes the limitations of traditional health monitoring, such as idealized assumptions of support conditions and the difficulty of using complex models. Gonzalez et al. [13] developed a finite element model to simulate vehicle–bridge interactions. This approach mitigates the interference of traffic loads on the dynamic characteristics of bridges. They proposed a stiffness damage assessment method based on forced frequency analysis. Almutairi et al. [14] used a one-dimensional convolutional neural network to evaluate structural stiffness damage. A cantilever beam model was used as an example. They successfully identified rotational stiffness damage at the supports under different excitation conditions. This provides a novel approach for assessing bridge boundary conditions. Existing health monitoring devices can monitor bridge stress and displacement in real-time using sensors. They can also issue alerts when risks are detected, allowing maintenance personnel to take emergency measures [15]. However, these sensors lack automated risk mitigation capabilities. When a bridge structure reaches a critical state, the alarm system usually provides a reaction time of several minutes to several hours. This duration depends on the location of the bridge, the type of risk involved, and the emergency response mechanism in place. Without immediate mechanical adjustments or active structural control, this reaction time is often inadequate to prevent major accidents. Especially in cases of instability at boundary nodes, relying solely on alarms may not effectively intervene before an incident occurs. This could lead to severe consequences, such as bridge overturning or beam dropping. Thus, it is clear that a single alarm function is insufficient. It cannot satisfy the safety needs of modern bridges operating in complex environments [16].

To address the shortcomings of existing bridge SHM in monitoring boundary nodes and managing risks, the process of beam bridge overturning was explored, and the key monitoring indicators for bridge overturning were identified. Based on the BRF monitoring indicators and self-locking theory, an adjustable intelligent force measurement bearing (AIFMB) was designed. By deriving its working mechanism, the corresponding structural design parameters were proposed. The mechanical characteristics of the bearing under triaxial loading were also analyzed. Finally, in the application of monitoring a real bridge, a specific three-span experimental bridge was utilized as a case study. This validated the effectiveness of the height adjustment and force measurement functions of the AIFMB in bridge monitoring. The results demonstrated that the AIFMB can accurately monitor dynamic changes in BRFs. Furthermore, it can autonomously adjust its structure to mitigate risks associated with boundary condition instabilities, such as girder overturning, pier settlement, or bearing unseating. These adjustments effectively prevent the progression of instability risks. The AIFMB addresses the critical gap in traditional SHM systems, which can monitor risks but lack the ability to implement emergency interventions. By combining precise monitoring with proactive control, the AIFMB significantly enhances the capability to manage overturning risks in bridges. This development provides a novel and effective solution to ensure the safe operation of bridges under complex working conditions.

## 2. Bridge Overturning Risk Analysis

### 2.1. Current Situation Analysis

In recent years, many countries have experienced multiple bridge collapse incidents. These accidents not only pose a serious threat to personal safety but also cause significant losses in public property. The frequent occurrence of these incidents reveals the vulnerability of bridge structures under load [17]. In practical engineering, bridge overturning and instability are among the most common and dangerous forms of structural failure. This issue must be given high priority.

The overturning and instability of bridges often stem from insufficient stability at boundary conditions, rather than inadequate strength or stiffness of the main beam itself. In recent years, many bridge overturning incidents in China (see Table 1) were primarily caused by structural instability and beam dropping. These issues arise from stability defects at boundary areas [18]. However, current bridge monitoring methods focus on parameters like stress, strain, and the deflection of the main beam. However, current bridge monitoring methods, such as monitoring parameters like stress, strain, and the deflection of the main beam, focus more on the overall stress state of the superstructure. Their effectiveness in controlling overturning risk is limited [19,20].

### 2.2. Overturning Mechanism Analysis

**Figure 1:**
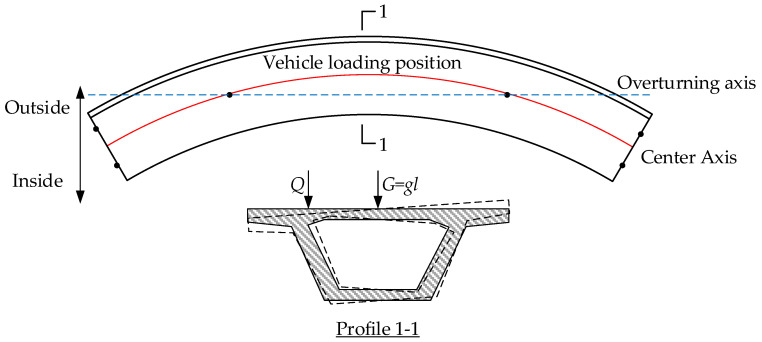
Schematic diagram of bridge overturning.

Under the action of moving loads, the superstructure of the bridge generates additional eccentric loading effects. When the value exceeds the effect of the self-weight, the superstructure will rotate around the overturning axis, leading to overturning instability (see Figure 1). Currently, the overturning stability coefficient method is widely used for design and safety assessment in engineering. This method treats the bridge superstructure as a rigid body and assumes that when the stability coefficient exceeds the limit, the bridge will overturn [26]. However, research has found that even if the bridge satisfies the stability coefficient requirements in design, overturning accidents can still occur. This happens when there is a lack of effective bearing connections between the piers and beams. Therefore, the traditional overturning stability coefficient method has limitations. It does not fully consider the impact of bearing conditions on bridge stability.

From actual cases of bridge overturning, it can be seen that bearing separation is the first critical state leading to bridge overturning. Bearing separation refers to the loss of contact between the superstructure of the bridge and the bearings, resulting in abnormal BRFs (such as negative values). In this condition, the structure is prone to rotational instability [27,28]. Therefore, monitoring BRFs becomes an important indicator for assessing the risk of bridge overturning. By monitoring changes in these forces, early signals of changes in boundary conditions can be captured before an overturning incident occurs. This allows for an effective evaluation of the stability of the bridge [29].

Although bearing conditions are crucial for bridge safety, existing research often focuses on cause analysis after overturning incidents occur. There is a lack of real-time warning systems and proactive prevention measures. Therefore, it is necessary to design an intelligent sensor that can monitor key parameters, such as BRFs, in real time. This sensor should take preventive measures in a timely manner before risks accumulate, thereby avoiding accidents.

## 3. Adjustable Intelligent Force Measurement Bearing Design

### 3.1. Structural Design

**Figure 2:**
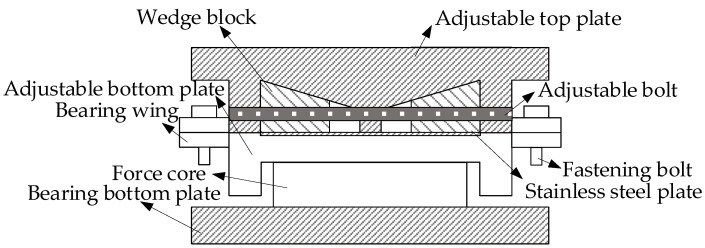
The AIFMB structure.

Bridge bearings are critical load-bearing components in the bridge support system. They balance and transfer the displacements and loads of the superstructure during the operation of the bridge [30]. The AIFMB adds a height adjustment sensor and a force measurement core, while replacing some of the original components. During its operation, the wedge blocks are securely self-locked between the upper and lower bearing plates to transfer the superstructure loads of the bridge. The height adjustment bolts connect the two wedge blocks to ensure the stability of the bearing (see Figure 2).

In the figure, the adjustable top plate is made of Q235 steel. Its upper surface connects with the bridge, while the lower surface is fitted with a stainless-steel plate that closely adheres to the wedge block. The AIFMB has bolt holes on both sides, which secure it to the adjustable bottom plate with bolts to prevent sliding along the bridge axis. The wedge block is also made of Q235 steel. Under lubricated conditions, the relative sliding between the wedge block and the adjustable top plate allows for the adjustment of the bearing height. The adjustable bolt is threaded with the wedge block. This arrangement ensures that the wedge block remains in a self-locking state, which is essential for the safe operation of the bearing. The fastening bolt is always kept tightened and must be removed when height adjustment is needed. Inside the force core, there are force sensors that monitor the force conditions of the bearing in real time. These sensors transmit data to a mobile device, providing an early warning of bridge collapse risks. The upper part of the bearing bottom plate supports the force core, while the lower part is closely connected to the pier and is fixed to the concrete with bolts. This configuration supports the upper loads and transfers them to the pier.

The force measurement function mainly relies on the force core, which monitors the bearing reaction force in real time. The force core measures 150 mm × 150 mm × 60 mm and includes a high-precision pressure sensor. With a sensitivity of 0.1% FS, it accurately measures the vertical load on the bearing, with a measurement range up to 500 kN. The working principle is based on the strain-resistance effect. When an external load acts on the bearing, the strain gauges within the core deform, causing a change in resistance. This change is then transmitted as real-time data through a data acquisition device. Additionally, the force core features temperature compensation, operating within a temperature range of −20 °C to 70 °C. This function eliminates data fluctuations caused by temperature effects, ensuring stability and reliability under various environmental conditions.

During use, the wedge block is securely self-locked between the upper and lower bearing plate. The two wedge blocks are connected by adjustable bolts to ensure their stability. During the height adjustment process, the twisting of the adjustable bolts causes the wedge blocks to slide relative to each other, enabling the adjustment of the bearing height. After the height adjustment is completed, the adjustable bolt and fastening bolt are secured, and temporary bearings are removed. This allows the entire load to be applied to the bearing, completing the entire height adjustment process.

### 3.2. Theoretical Formula Derivation

**Figure 3:**
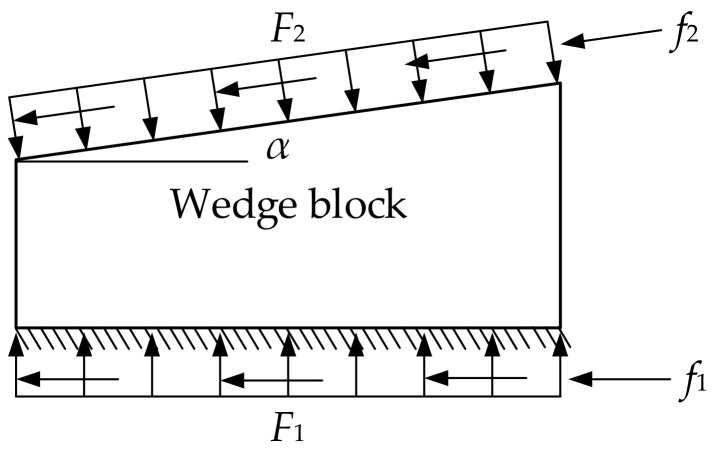
Force analysis of wedge block.

When the superstructure exerts pressure on the wedges, it creates a tendency for the wedges to move horizontally. This results in friction. As a result, there can be a situation where, regardless of the magnitude of the applied external force, the wedges cannot slide (see Figure 3). This phenomenon is known as mechanical self-locking [31,32].

The friction coefficients of the contact surfaces are denoted as *µ*_1_ and *µ*_2_. The pressures exerted on the wedge block by the two components are *f*_1_ and *f*_2_, and the corresponding friction forces are *F*_1_ and *F*_2_.

(1) $$F_{2}\cos\alpha +f_{2}\sin\alpha =F_{1},$$

(2) $$F_{2}\sin\alpha =f_{2}\cos\alpha +f_{1},$$

(3) $$\left\{\begin{array}{l} f_{2}=\mu_{2}F_{2}\\ f_{1}=\mu_{1}F_{1} \end{array}\right..$$

where, *α* is the angle of the wedge block.

By combining the above equations, the critical value of the self-locking angle can be obtained:

(4) $$\alpha =\arctan\frac{\mu_{1}+\mu_{2}}{1-\mu_{1}\mu_{2}},$$

(5) $$\alpha =\arctan\mu_{1}+\arctan\mu_{2}.$$

Since *µ*_1_ = *µ*_2_ = *µ*, the following equation holds:

(6) α≤2arctanμ.

Therefore, when α ≤ 2arctan*µ*, the wedge will self-lock.

When the bearing height is lowered, the bolt applies an opposite pulling force to the wedge block. The wedge block needs to overcome the frictional forces between the adjustable top plate and bottom plate, causing it to slide [33].

**Figure 4:**
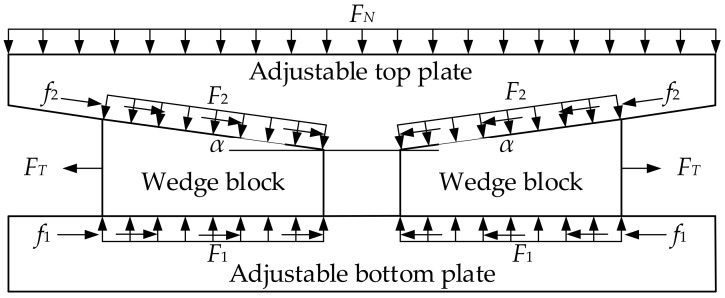
Bearing force analysis.

Similarly, when the bearing height is lifted, the wedge block not only needs to overcome the frictional forces between the adjustable top and bottom plate, but also the horizontal pressure exerted by the adjustable top plate on the wedge block (see Figure 4).

When the bearing height is lowered, the following applies:

(7) $$F_{N}=2{F^{\prime }}_{2}\cos\alpha +2{f^{\prime }}_{2}\sin\alpha ,$$

(8) $$F_{2}\cos\alpha +f_{2}\sin\alpha =F_{1},$$

(9) $$F_{T}=F_{2}\sin\alpha +f_{2}\cos\alpha +f_{1}.$$

Since *f*_1_ = *µ*_1_*F*_1_, *f*_2_ = *µ*_2_*F*_2_, *F*_2_^’^ = *F*_2_, and *f*_2_^’^ = *f*_2_, the following applies:

(10) $$F_{T}=\frac{1}{2}\left(\frac{\mu_{1}-\tan\alpha }{1+\mu_{1}\tan\alpha }+\mu_{2}\right)F_{N}.$$

where, *F_N_* is the pressure applied to the adjustable top plate and *F_T_* is the force required to overcome friction and initiate sliding.

When the bearing height is lifted, the following applies:

(11) $$F_{N}=2{F^{\prime }}_{2}\cos\alpha -2{f^{\prime }}_{2}\sin\alpha ,$$

(12) $$F_{2}\cos\alpha -f_{2}\sin\alpha =F_{1},$$

(13) $$F_{T}=F_{2}\sin\alpha +f_{2}\cos\alpha +f_{1}.$$

Since *f*_1_ = *µ*_1_*F*_1_, *f*_2_ = *µ*_2_*F*_2_, *F*_2_^’^ = *F*_2_ and *f*_2_^’^ = *f*_2_, the following applies:

(14) $$F_{T}=\frac{1}{2}\left(\frac{\mu_{2}+\tan\alpha }{1-\mu_{2}\tan\alpha }+\mu_{1}\right)F_{N}.$$

Additionally, since the coefficients of friction on the contact surfaces are the same, the required tensile force when the bearing height is lowered is as follows:

(15) $$F_{T}=\frac{1}{2}\left(\frac{\mu -\tan\alpha }{1+\mu \tan\alpha }+\mu \right)F_{N}.$$

The required tensile force when the bearing is lifted is as follows:

(16) $$F_{T}=\frac{1}{2}\left(\frac{\mu +\tan\alpha }{1-\mu \tan\alpha }+\mu \right)F_{N}.$$

Therefore, when the wedge angle α > 2arctan*µ*, mechanical self-locking is not achieved. As a result, the wedge block will slide to both sides under the compression of the adjustable top plate and bottom plate.

**Figure 5:**
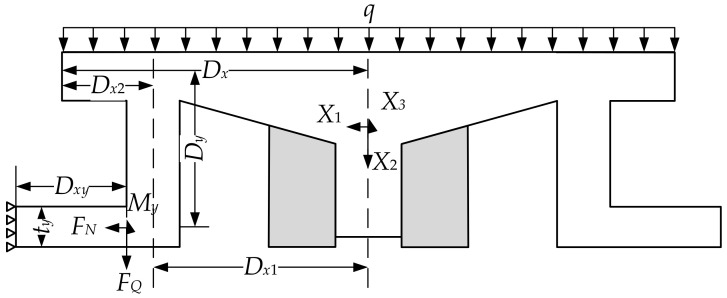
Wedge block force analysis.

The bearing is a symmetric structure, so the adjustable top plate can be analyzed as a half structure. Thus, the symmetry axis of the adjustable top plate can be simplified to a vertical sliding bearing, which has horizontal axial force and torsional constraints. Since stainless steel plates are embedded on the upper and lower surfaces of the wedge block, the friction force can be ignored. This allows the wedge block to be simplified to a horizontal sliding bearing. At this point, the half structure can be regarded as a three-degree-of-freedom hyperstatic structure (see Figure 5).

The axial force, shear force, and bending moment under fixed constraints are denoted as *X*_1_, *X*_2_, and *X*_3_, respectively. According to the force method equation, the following applies:

(17) $$\left\{\begin{array}{l} \delta_{11}X_{1}+\delta_{12}X_{2}+\delta_{13}X_{3}+\Delta_{1P}=0\\ \delta_{21}X_{1}+\delta_{22}X_{2}+\delta_{23}X_{3}+\Delta_{2P}=0\\ \delta_{31}X_{1}+\delta_{32}X_{2}+\delta_{33}X_{3}+\Delta_{3P}=0 \end{array}\right.,$$

(18) $$X_{1}=\frac{\left|\begin{array}{ccc} \Delta_{1P} & \delta_{12} & \delta_{13}\\ \Delta_{2P} & \delta_{22} & \delta_{23}\\ \Delta_{3P} & \delta_{32} & \delta_{33} \end{array}\right|}{\left|\begin{array}{ccc} \delta_{11} & \delta_{12} & \delta_{13}\\ \delta_{21} & \delta_{22} & \delta_{23}\\ \delta_{31} & \delta_{32} & \delta_{33} \end{array}\right|};X_{2}=\frac{\left|\begin{array}{ccc} \delta_{11} & \Delta_{1P} & \delta_{13}\\ \delta_{21} & \Delta_{2P} & \delta_{23}\\ \delta_{31} & \Delta_{3P} & \delta_{33} \end{array}\right|}{\left|\begin{array}{ccc} \delta_{11} & \delta_{12} & \delta_{13}\\ \delta_{21} & \delta_{22} & \delta_{23}\\ \delta_{31} & \delta_{32} & \delta_{33} \end{array}\right|};X_{3}=\frac{\left|\begin{array}{ccc} \delta_{11} & \delta_{12} & \Delta_{1P}\\ \delta_{21} & \delta_{22} & \Delta_{2P}\\ \delta_{31} & \delta_{32} & \Delta_{3P} \end{array}\right|}{\left|\begin{array}{ccc} \delta_{11} & \delta_{12} & \delta_{13}\\ \delta_{21} & \delta_{22} & \delta_{23}\\ \delta_{31} & \delta_{32} & \delta_{33} \end{array}\right|}.$$

Through the calculations of the statically determinate structure, the axial force *F_N_*, shear force *F_Q_*, and bending moment *M_y_* can be obtained:

(19) $$\left\{\begin{array}{l} F_{Q}=qD_{X_{2}}-X_{2}\\ F_{N}=X_{1}\\ M_{y}=D_{y}X_{1}-D_{X_{2}}X_{2}+X_{3}-\frac{1}{2}q\left(D_{X_{1}}^{2}-D_{X_{2}}^{2}\right) \end{array}\right..$$

Thus, the maximum shear stress is as follows:

(20) $$\tau_{\max}=\frac{3F_{Q}}{2bh}.$$

Thus, the maximum shear stress in the bearing wing is as follows:

(21) $$\tau_{\max}=\frac{3\left(qD_{x_{2}}-X_{2}\right)}{2bh}.$$

Under unilateral bending load conditions, the following conditions must be satisfied:

(22) $$M_{y}\leq W_{pnx}f.$$

where, *M_y_* is the bending moment about the y-axis of the beam section, and *W_pnx_* is the plastic net section modulus about the x-axis.

By combining Equations (17)–(22), the following can be obtained:

(23) $$\frac{D_{y}X_{1}-D_{X_{2}}X_{2}+X_{3}-\frac{1}{2}q\left(D_{X_{1}}^{2}-D_{X_{2}}^{2}\right)}{W_{pnx}}\leq f.$$

where, *q* is the line load; *D_y_* is the length of the stainless-steel plate; *D_X_*_1_ is the distance from the centerline of the baffle to the axis of symmetry; *D_X_*_2_ is the distance from the flange of the upper bearing plate to the centerline of the baffle.

At this time, the normal stress on the section is as follows:

(24) $$\sigma =\frac{F_{N}}{A}.$$

where, *σ* is the contact area between the adjustable top plate and the wing plate.

At the edge where the wing plate connects to the adjustable top plate, it will simultaneously experience significant normal stress, shear stress, and localized compressive stress.

(25) $$\sqrt{\sigma^{2}+\sigma_{c}^{2}-\sigma \sigma_{c}+3\tau^{2}}\leq \beta_{1}f.$$

where, *σ*, *σ_c_*, and *τ* denote the bending normal stress, localized compressive stress, and shear stress, respectively. *β*_1_ is the strength design value amplification factor for the reduced stress verification.

By combining the above equations, we obtain the following:

(26) $$\sqrt{\begin{array}{l} {\left(\frac{F_{N}}{A}\right)}^{2}+{\left[\frac{D_{y}X_{1}-D_{X_{2}}X_{2}+X_{3}-\frac{1}{2}q\left(D_{X_{1}}^{2}-D_{X_{2}}^{2}\right)}{W_{pnx}}\right]}^{2}\\ -\left(\frac{F_{N}}{A}\right)\left[\frac{D_{y}X_{1}-D_{X_{2}}X_{2}+X_{3}-\frac{1}{2}q\left(D_{X_{1}}^{2}-D_{X_{2}}^{2}\right)}{W_{pnx}}\right]+27{\left[\frac{\left(qD_{x_{2}}-X_{2}\right)}{2bh}\right]}^{2} \end{array}}\leq \beta_{1}f,$$

(27) $$N_{v}^{b}\geq n_{v}\frac{\pi d^{2}}{4}f_{v}^{b}$$

where, *n_v_* is the number of shear surfaces for each bolt; *d* is the diameter of the bolt; *f_v_^b^* is the design value of the bolt’s shear strength; and *N_v_^b^* is the design value of the bolt’s shear bearing capacity.

### 3.3. Ultimate Strength Analysis

**Figure 6:**
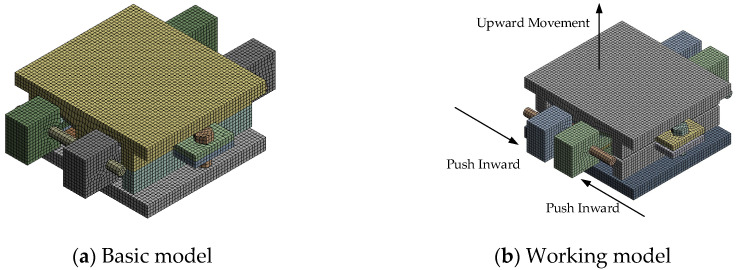
Finite element model of the AIFMB.

The finite element model of the AIFMB is established using the nonlinear finite element software ANSYS v10.0. The model is divided into 80,744 elements, with the element type chosen as solid185 hexahedral elements. The material parameters are shown in Table 2, and the finite element model is shown in Figure 6.

The adjustable top plate, adjustable bottom plate, and wedge are in contact through stainless steel and polytetrafluoroethylene materials. Therefore, the contact definition between components is set as Frictional contact, with a friction coefficient of 0.03. The contact between the adjustable bottom plate and the bearing core is defined as bonded contact. Considering that the contact surfaces among the bonded bottom plate, bearing core, force-bearing body, and cavity need to restrict friction, the component contact is defined as bonded contact. The contact between the threads and threaded holes is not considered; thus, the component contact is simplified and defined as bonded contact.

**Figure 7:**
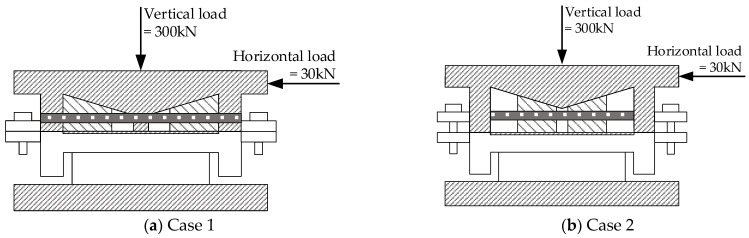
Loading conditions.

To ensure the safety performance of the AIFMB, the bearing must be able to withstand 1.5 times its design load when at maximum working height. This ensures a substantial safety margin during operation. Therefore, based on the load capacity analysis results, two unfavorable loading conditions are considered (see Table 3 and Figure 7).

**Figure 8:**
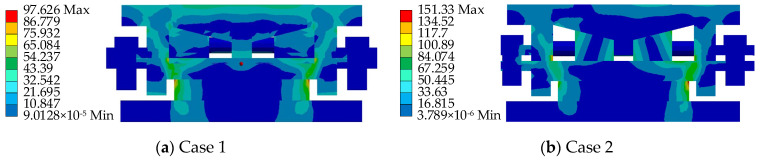
The AIFMB stress.

**Figure 9:**
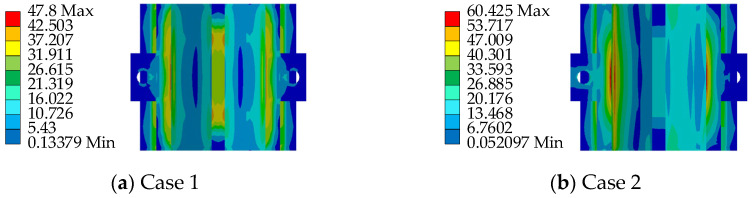
The adjustable top plate stress.

**Figure 10:**
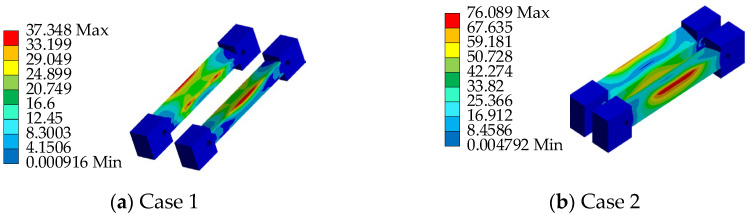
The wedge block stress.

**Figure 11:**
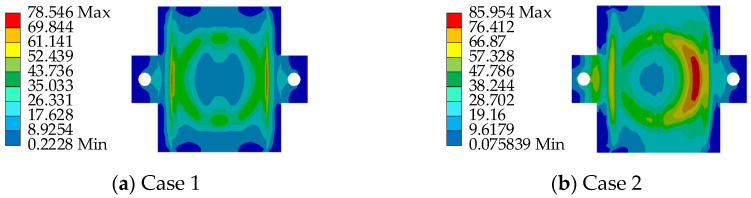
The adjustable bottom plate stress.

Based on the aforementioned loading conditions, a performance analysis of the AIFMB is conducted (see Figure 8, Figure 9, Figure 10, Figure 11).

The figures indicate that the stress in the contact area between the adjustable top plate and the wedge block is significantly higher than in other areas, which aligns with expected mechanical behavior. The stress distribution in the force core is relatively uniform, with slightly higher stress values observed in the outer region compared to the central area. Once the bearing begins height adjustment, stress concentration appears to varying degrees on both the adjustable top and bottom plates. This occurs because the change in height alters the load transfer characteristics of the bearing. The stress concentration in the contact area of the wedge block is caused by the change of the joint force between the wedge block and the bottom plate. Under the most unfavorable working conditions, the maximum stress of the support is 151.33 MPa. This value remains below the allowable compressive stress of 207 MPa for stainless steel, meeting the design requirements.

## 4. Field Application of AIFMB in Bridge Monitoring

### 4.1. Bridge Profile

The study focuses on a three-span continuous steel box girder experimental bridge located at the Tsinghua University Bridge Laboratory. This experimental bridge was designed and constructed at a 1:7 scale based on an existing operational bridge. The bridge has three spans with a total length of 33 m, comprising spans of 9 m, 15 m, and 9 m, with a width of 2.25 m, arranged in an orthogonal layout.

**Figure 12:**
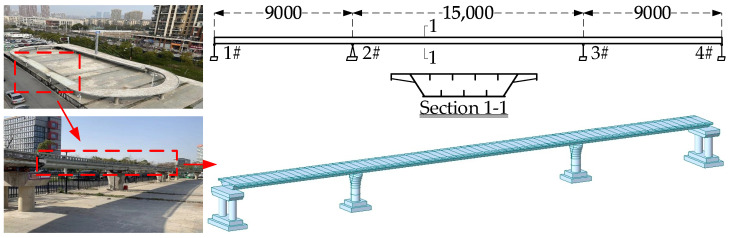
Finite element model of the bridge.

**Figure 13:**
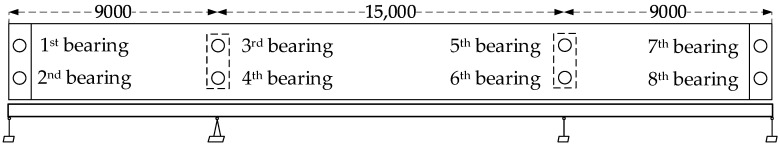
The layout of the bearings.

The superstructure consists of a uniformly high steel box girder, using Q345 steel and C30 concrete. Both the top and bottom plates are reinforced with stiffeners, with dimensions of 100 mm × 10 mm. The spacing for the stiffeners on the top plate is 400 mm, while on the bottom plate, it is 350 mm. A finite element model of the three-span continuous girder bridge was established based on the dimensions of the experimental bridge (see Figure 12). The layout of the bearings is shown in Figure 13.

### 4.2. Experimental Condition

**Figure 14:**
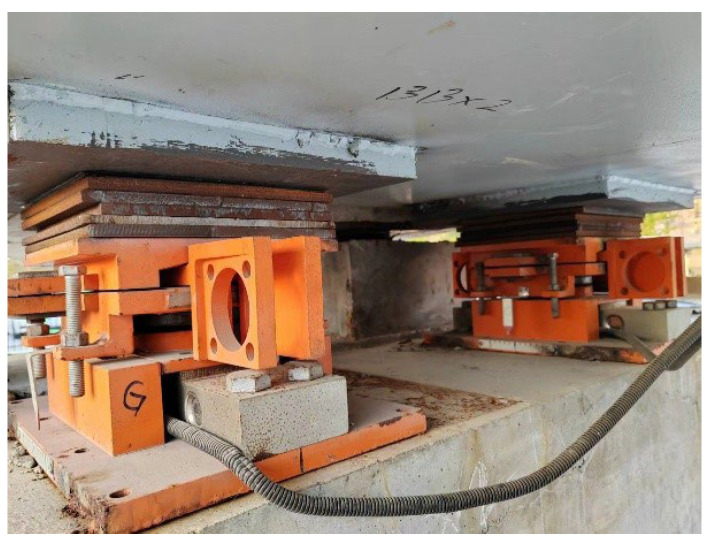
AIFMB installation.

To verify the effectiveness of the AIFMB in monitoring the actual bridge, the original bearings of the experimental bridge were replaced with the AIFMB (see Figure 14). Additionally, adjustments were made to ensure compatibility between the bearings and the bridge structure. This guarantees that the new bearings can effectively utilize their height adjustment and force measurement features.

The primary objective of the experiment is to verify the force measurement and height adjustment functions of the AIFMB. The specific experimental conditions are detailed in Table 4. When verifying the force measurement function of AIFMB, no adjustment is made to any bearing. When verifying its adjustment function, only the fourth bearing is adjusted by 0.2 mm to 1.2 mm, while other bearings remain unchanged.

**Figure 15:**
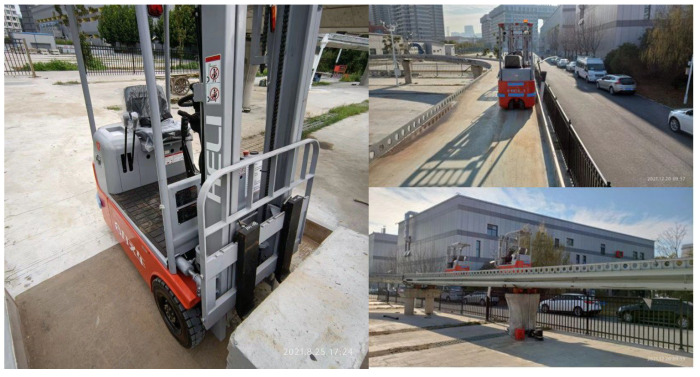
Field experimentation.

In the experiment, the loading method used was a moving load. Concrete weights were attached to the experimental forklift to simulate a moving load of 20 kN. This setup aimed to analyze the performance of the AIFMB under different loading conditions. It is noteworthy that the wheelbase of the forklift is 1 m, and the distance between the front wheels is 0.8 m. Due to its relatively small wheelbase and track width, it can be approximated as a concentrated load, assuming that it does not induce vehicle axle effects (see Figure 15).

### 4.3. Application Result Analysis

#### 4.3.1. Force Measurement Result

**Figure 16:**
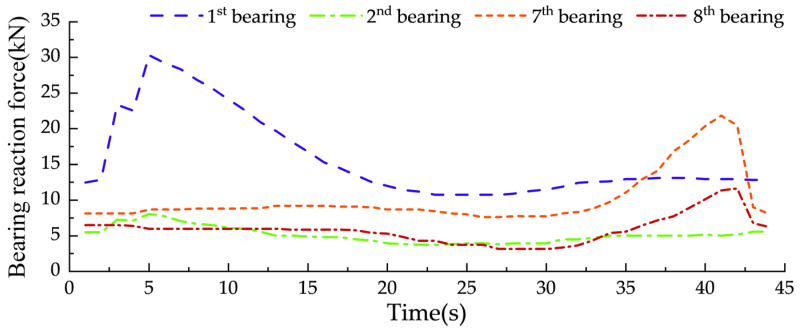
Beam end BRF data under vehicle load.

To validate the force measurement function of the bearing, the moving load was applied according to the Case normal condition. The BRF data presented in Figure 16 were collected in real time during a single loading experiment. No averaging or smoothing algorithms were applied to the data, ensuring that the results directly reflect the dynamic behavior of the bearings under the experimental conditions.

From Figure 16, the BRFs of the first and second bearings show a significant difference during the initial phase of vehicle loading (around the 5-s mark). This difference is caused by the narrow cross-sectional width of the scaled bridge and its ring-shaped structure. When the vehicle enters the bridge, it is not fully centered, creating an eccentric loading effect (refer to Figure 15). As a result, the peak BRF at the first bearing is 30 kN, while the peak BRF at the second bearing is 7 kN. As the vehicle moves forward, its path aligns with the bridge centerline, reducing the eccentric loading effect. Finally, the peak BRFs at the seventh and eighth bearings are 22 kN and 12 kN, respectively.

The dynamic fluctuations of the BRF can be effectively sensed by the force core as the experimental forklift moves. Additionally, the variations in reaction forces for different supports also differ, which aligns with the characteristic patterns of the BRFs. At the moment, when the experimental forklift enters and exits the bridge, there will be a noticeable sudden change in the BRF. This phenomenon is primarily caused by the impact effect of the vehicle. It can be concluded that the sensitivity of the AIFMB for force measurement meets the requirements for recognizing vehicle speed and axle spacing. This demonstrates its effectiveness and reliability in practical applications.

#### 4.3.2. Height Adjustment Results

**Figure 17:**
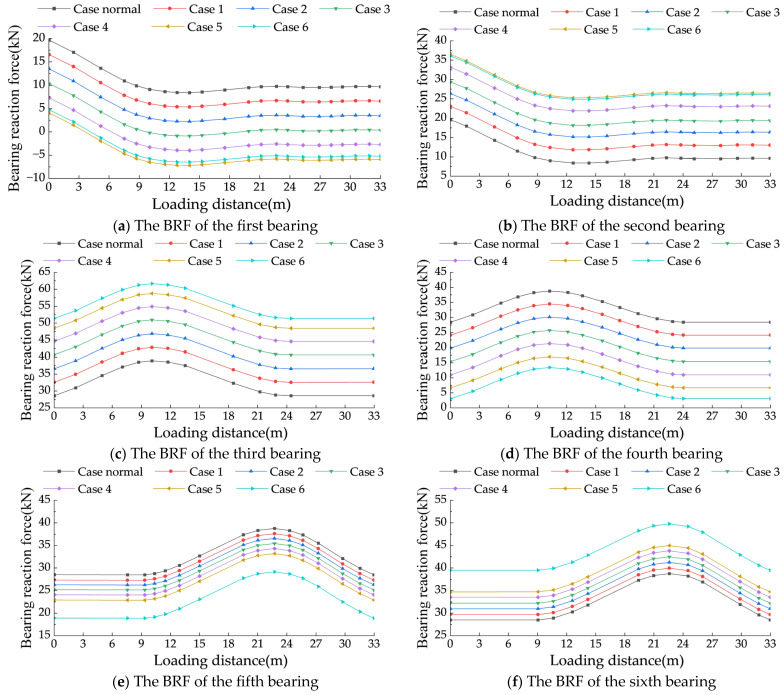

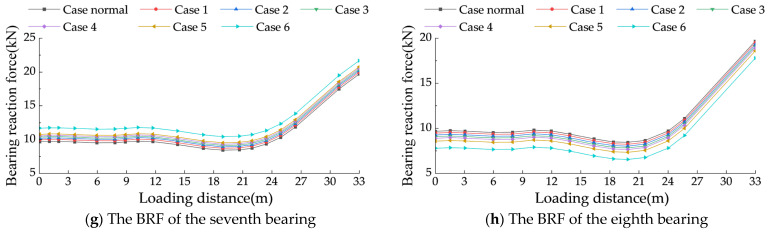
Reaction force variations of each bearing under different case conditions.

Furthermore, height adjustments for the fourth bearing were conducted under Cases 1 to 6. A moving load of 20 kN was applied to simulate the movement of a vehicle on the bridge deck. The impact of different height adjustments on the AIFMB was analyzed (see Figure 17).

The BRF at the mid span is significantly affected by the height adjustment. After a 1.2 mm adjustment, the bearing reaction varies by approximately 40 kN. In contrast, the BRF at the side spans shows less variation. The reaction force variations for the seventh and eighth bearings after a height adjustment of 1.2 mm are 15 kN. The reaction forces of the first, fourth, fifth, and eighth bearings decrease as the height adjustment increases. In contrast, the reaction forces of the second, third, sixth, and seventh bearings increase with the rise in height adjustment. It is noteworthy that when the height of the fourth bearing is adjusted by 1.2 mm, it approaches a state of being nearly unseated. At this point, further increases in height have limited effects on the reaction forces of the other bridge pier bearings.

The experimental results demonstrate that adjusting the height of the fourth bearing significantly affects the reaction forces of the bridge bearings, although the extent of this influence varies depending on the bearing location. For bearings in the side spans (first, second, seventh, and eighth bearings), which are farther from the adjustment point, changes in reaction forces are primarily constrained by the global stiffness of the bridge. This constraint suppresses the magnitude of the changes, often keeping them smaller than the resolution range of the sensors. As a result, the force–response curves for these bearings tend to overlap. In contrast, mid-span bearings (third, fifth, and sixth bearings), located closer to the adjustment point, are more sensitive to local height changes. This sensitivity leads to a pronounced nonlinear increase in reaction forces. As the height adjustment of the fourth bearing progresses in Case 5 and Case 6, the bearing nears a state of separation. Beyond this point, further adjustments have minimal impact on the reaction forces of other pier bearings. The reaction forces stabilize, indicating a saturation effect in the adjustment process.

## 5. Discussion

This study developed the AIFMB and experimentally validated its performance in real-time monitoring and active adjustment. The results demonstrate that the AIFMB effectively monitors changes in BRFs and improves load distribution at boundary nodes to mitigate instability risks through height adjustment functionality. However, compared to recent advances in SHM, there are still shortcomings in the evaluation of bridge stiffness and overall health. Further enhancements are necessary to address these gaps.

1. Future research could combine real-time eigen perturbation techniques by introducing vibration modal parameter analysis. This method would improve the ability of the device to detect changes in bridge stiffness. Adding modal parameter changes to bearing reaction force data would enable the monitoring of both global and local risks. This approach can help address the current lack of an overall health assessment.
2. The AIFMB now uses bearing reaction forces as the only monitoring parameter. This approach makes it hard to identify multiple risk sources under complex conditions. Future work could include multi-modal data, such as vibration, temperature, and displacement. This combination of data will improve adaptability and accuracy when analyzing risks in complex scenarios.
3. To improve early warnings for instability and potential risks, new prediction modules based on historical reaction forces and modal data should be developed. Computer vision and deep learning can be used to analyze historical data and predict long-term changes in bridge performance. This method would improve the overall monitoring and prediction abilities of the device.

## 6. Conclusions

To mitigate the risk of bridge overturning under heavy vehicle eccentric loading, this paper addresses the monitoring and risk management needs at the boundary nodes of the bridge. It derives the overturning process of beam bridges and proposes a method to characterize the unseating state of bearings using BRF indicators. Furthermore, the AIFMB is designed and developed. The working mechanism and bearing capacity of this bearing are derived and analyzed based on the principle of self-locking, confirming the feasibility of the intelligent bearing. Finally, taking a certain experimental bridge as an example, the height adjustment and force measurement functions of the AIFMB are evaluated, leading to the following conclusions:

1. The derived relationships between lifting force-vertical force and horizontal displacement-vertical displacement validate the feasibility of the AIFMB structure. Under the design load, the stresses of each component remain within reasonable strength design limits under adverse conditions. This indicates that the new bearing design has an effective structure and accurate parameter selection.
2. The AIFMB accurately measures the changes in bearing reaction force caused by the test vehicle and sensitively detects the instantaneous impacts. This verifies that its sensitivity in force measurement meets the requirements for vehicle speed and axle spacing identification. This new bearing can monitor abnormal bearing reactions in real time, ensuring the safe operation of the bridge.
3. The moving load is sensitive to the settlement of the bridge piers, and the reaction force at the mid-span bearing is significantly affected by the settlement. After the AIFMB is raised, the reaction force of the bridge bearings approaches normal values, leading to safer and more reasonable stress distribution in the bridge. The AIFMB effectively addresses the issue of bridge pier settlement.
4. Future work could enhance the device by integrating real-time eigen perturbation techniques to improve its sensitivity to bridge stiffness changes. Combining modal parameter analysis with bearing reaction force data could improve the monitoring of both global and local risks. This approach would help address current limitations in comprehensive health assessments.

## Figures and Tables

**Table 1:** Recent accidents of overturning accidents on the Chinese mainland.

Date	Location	Accident	Consequence
23 October 2007	Minzu East Road Viaduct, Baotou City, Nei Mongol, China	Main girder of the viaduct suddenly overturned [21]	Many people were injured
15 July 2009	Shanxi-Tianjin Expressway, Tianjin City, China	Ramp bridge A overturned [21]	6 dead and 4 injured
26 November 2010	Rapid Inner Ring West Line, Nanjing City, Jiangsu, China	Side tumbling of steel box beam [18]	7 dead and 3 injured
21 February 2011	Chunhui Interchange, Shangyu City, Zhejiang, China	On-ramp collapsed [22]	3 injured
24 August 2012	Qunli Viaduct, Harbin City, Heilongjiang, China	Overall overturning of approach bridge box girder [23]	3 dead and 5 injured
19 June 2015	Guangdong-Jiangxi Expressway, Heyuan City, Guangdong, China	Ramp bridge collapsed [24]	1 dead and 4 injured
23 May 2016	Center Line Viaduct, Shanghai City, China	Damage on bridge surface [25]	No casualties

**Table 2:** Material parameters.

Category	Elasticity Modulus	Poisson Ratio	Size (mm)	Thickness (mm)
Adjustable top plate	2.1 × 10^5^ MPa	0.3	240 × 215	20
Adjustable bottom plate			190 × 215	20
Wedge block			40 × 215	10–20
Adjustable bolt			R20	\

**Table 3:** Unfavorable loading conditions.

Loading Conditions	Detail	Vertical Load (kN)	Horizontal Load (kN)
Case 1	Basic model	300	30
Case 2	Working model	300	30

**Table 4:** Experimental condition.

Conditions	Detail	Height Adjustment Position	Height Adjustment Degree
Case normal	Force measurement	/	/
Case 1	Height adjustment	Fourth bearing	0.2 mm
Case 2			0.4 mm
Case 3			0.6 mm
Case 4			0.8 mm
Case 5			1.0 mm
Case 6			1.2 mm

## Data Availability

The data presented in this study are available on request from the corresponding author. The data are not publicly available as research is continuing.
